# Myeloid-derived suppressor cell subtypes differentially influence T-cell function, T-helper subset differentiation, and clinical course in CLL

**DOI:** 10.1038/s41375-021-01249-7

**Published:** 2021-05-02

**Authors:** Gerardo Ferrer, Byeongho Jung, Pui Yan Chiu, Rukhsana Aslam, Florencia Palacios, Andrea Nicola Mazzarello, Stefano Vergani, Davide Bagnara, Shih-Shih Chen, Sophia Yancopoulos, Aliki Xochelli, Xiao-Jie Yan, Jan A. Burger, Jacqueline C. Barrientos, Jonathan E. Kolitz, Steven L. Allen, Kostas Stamatopoulos, Kanti R. Rai, Barbara Sherry, Nicholas Chiorazzi

**Affiliations:** 1 Karches Center for Oncology Research, The Feinstein Institutes for Medical Research, Northwell Health, Manhasset, NY USA; 2 Center for Immunology & Inflammation, The Feinstein Institutes for Medical Research, Northwell Health, Manhasset, NY USA; 3 Institute of Applied Biosciences, CERTH, Thessaloniki, Greece; 4 Hematology Department and HCT Unit, G. Papanicolaou Hospital, Thessaloniki, Greece; 5 Department of Leukemia, Division of Cancer Medicine, The University of Texas MD Anderson Cancer Center, Houston, TX USA; 6 Department of Medicine, Northwell Health, Manhasset, NY USA; 7 Department of Medicine, Donald and Barbara Zucker School of Medicine at Hofstra/Northwell, Hempstead, NY USA; 8 Department of Molecular Medicine and Surgery, Karolinska Institutet, Stockholm, Sweden; 9 Department of Molecular Medicine, Donald and Barbara Zucker School of Medicine at Hofstra/Northwell, Hempstead, NY USA

**Keywords:** Cancer microenvironment, Tumour immunology

## Abstract

Cancer pathogenesis involves the interplay of tumor- and microenvironment-derived stimuli. Here we focused on the influence of an immunomodulatory cell type, myeloid-derived suppressor cells (MDSCs), and their lineage-related subtypes on autologous T lymphocytes. Although MDSCs as a group correlated with an immunosuppressive Th repertoire and worse clinical course, MDSC subtypes (polymorphonuclear, PMN-MDSC, and monocytic, M-MDSCs) were often functionally discordant. In vivo, PMN-MDSCs existed in higher numbers, correlated with different Th-subsets, and more strongly associated with poor clinical course than M-MDSCs. In vitro, PMN-MDSCs were more efficient at blocking T-cell growth and promoted Th17 differentiation. Conversely, in vitro M-MDSCs varied in their ability to suppress T-cell proliferation, due to the action of TNFα, and promoted a more immunostimulatory Th compartment. Ibrutinib therapy impacted MDSCs differentially as well, since after initiating therapy, PMN-MDSC numbers progressively declined, whereas M-MDSC numbers were unaffected, leading to a set of less immunosuppressive Th cells. Consistent with this, clinical improvement based on decreasing CLL-cell numbers correlated with the decrease in PMN-MDSCs. Collectively, the data support a balance between PMN-MDSC and M-MDSC numbers and function influencing CLL disease course.

## Introduction

In chronic lymphocytic leukemia (CLL), a CD5^+^ B-cell progressively expands and accumulates in the bone marrow and secondary lymphoid organs [1–4]. At these sites, CLL cells engage in complex, incompletely defined interactions with other cell types such as non-leukemic T cells, myeloid cells, and mesenchymal stromal cells that are critical for survival and proliferation of the leukemia [5, 6].

Immune disturbance is a common feature of CLL [7]. Alterations in T-cell numbers and function occur, involving impaired immune synapse formation due to T-cell cytoskeletal dysfunction and increased expression of inhibitory checkpoint molecules [8–11]. T cells are a main target of myeloid-derived suppressor cells (MDSCs), the latter a heterogeneous population divisible into monocyte-like (CD14^+^, “M-MDSC”) and polymorphonuclear-like (CD15^+^, “PMN-MDSC”) subsets [12]. Notably, both types can block T-cell growth [12] and modulate T-cell differentiation into functional T-helper (Th)-cell subsets [13]. MDSCs promote the progression of many cancers by perturbing anti-tumor immune function and allowing tumor cells to escape immune surveillance. Although MDSC numbers in the periphery positively correlate with solid tumor counts, it is within the tumor microenvironment (TME) that suppressor function is strongest [14, 15]. In CLL, M-MDSCs can be elevated in patients with higher-risk disease features [16–18].

Major advances in the treatment of CLL have occurred with the use of molecules targeting stimulatory pathways [19]. In particular, ibrutinib, an orally administered Bruton’s tyrosine kinase (BTK) inhibitor, blocks B-cell receptor and chemokine- and cytokine-receptor pathways, thereby affecting survival and expansion of CLL cells [19]. However because other cell types express BTK and because ibrutinib can bind to other members of the Tec kinase family, the impact of the drug on the entire complement of cells involved in the disease is not entirely understood.

We have investigated the connection between MDSCs and T cells and the extent that ibrutinib alters these interactions. We observed that CLL patients have significantly higher numbers of MDSCs, particularly PMN-MDSCs. PMN-MDSCs suppressed T-cell growth, whereas M-MDSCs were impaired in this action, due to the influence of TNFα. Furthermore, although MDSCs as a group strongly associated with specific immunosuppressive Th-cell populations in vivo, M-MDSCs more effectively promoted Th1- and Th2-cell differentiation in vitro. Finally, ibrutinib reduced significantly the numbers of MDSCs and PMN-MDSCs and altered MDSC-induced differentiation of autologous naive T (T_N_) cells toward Th1 and away from Th2 cells, thereby leading to a more protective, anti-leukemia TME.

## Materials and methods

### Patients

Cryopreserved PBMCs from 75 CLL patents diagnosed according to iwCLL criteria [20] were studied (Table S1). These patients were cared for at Northwell Health, New Hyde Park, New York (*n* = 34); at the Institute of Applied Biosciences, G. Papanicolaou Hospital, Thessaloniki, Greece (*n* = 21); and at MD Anderson Cancer Center, Houston, Texas (*n* = 20). PBMCs from each institution were separated by density gradient centrifugation using Ficoll Paque/Hypaque and cryopreserved in RPMI-1640 medium supplemented with heat-inactivated fetal bovine serum (FBS) and 10% of DMSO until use. Samples were collected from 55 untreated patients at diagnosis and from 20 before and during ibrutinib therapy. In addition, samples from 17 age- and gender-matched healthy individuals served as controls (HCs). Written informed consent was received from participants prior to enrollment in accordance with the Helsinki Declaration and as approved by the Institutional Review Boards of each institution.

### Effect of MDSCs on T-cell proliferation

FACS was used to isolate T cells, PMN-MDSCs, M-MDSCs, and classical monocytes based on the following respective membrane phenotypes: CD3^+^CD19^−^; HLA-DR^lo^CD11b^+^CD33^+^CD15^+^; HLA-DR^lo^CD11b^+^CD33^+^CD14^+^ and (CD11b^+^CD14^+^HLA-DR^+^). To measure MDSC-induced suppression of autologous T-lymphocyte proliferation, after exposing T cells to carboxyfluorescein diacetate succinimidyl ester (CFDA-SE 1 µM; ThermoFisher) for 10 min at 37 °C, they were cultured with or without MDSCs in a 2:1 ratio, respectively. T-cell proliferation was measured by the extent of CFDA-SE dilution using a BD LSRII cytometer. Cultures were carried out in complete medium consisting of RPMI1640 (Gibco), 10% FBS (Atlanta Biologicals), 1× penicillin–streptomycin (GE Healthcare Life Sciences), GlutaMAX (Gibco) supplemented with human IL-2 (50 µ/ml; R&D), and anti-CD3/CD28 beads (Thermo Scientific) according to the manufacture’s recommendation for 72 h.

### In vitro induction of M-MDSCs

Induced M-MDSCs (iM-MDSCs) were generated from CLL autologous myeloid precursors with a protocol modified from Lechner et al. [21]. After enriching CD33^+^ cells from PBMCs using antihuman CD33 MicroBeads (Miltenyi Biotec), cells (1 × 10^5^ cells/ml) were suspended in complete culture medium supplemented with IL-6 (10 ng/ml; R&D Systems), IL-10 (10 ng/ml; R&D Systems) and GM-CSF (10 ng/ml; R&D Systems), and then cultured for 7 days in a Nunc UpCell 12 MultiDish (Thermo Scientific). In some instances, TNFα (20 ng/ml; R&D Systems) was added at the initiation of culture. After the incubation period, adherent cells were washed and collected from Nunc UpCell 12 MultiDish by cooling plates to below 30 °C, thereby making the culture dish surfaces hydrophobic and detaching adherent cells without altering surface molecule expression [22].

### In vitro Th-cell differentiation

Ten thousand FACS-isolated, CD4^+^ T_N_ cells (CD3^+^CD4^+^CD62L^+^CD45RO^−^) were cultured without or with 20 × 10^3^ M-MDSCs, PMN-MDSCs, or monocytes plus anti-CD3/CD28 beads and IL-2. After 7 days, cells and supernatants were collected. The latter were frozen for subsequent batch analyses; the former were stimulated for 4.5 h with PMA (10 ng/ml; EMD Millipore) and ionomycin (250 ng/ml; EMD Millipore) in the presence of the Golgi inhibitor, monensin (1/1500; BD Bioscience). Finally, cells were exposed to appropriate fluorochrome-labeled mAbs, and membrane expression analyzed by flow cytometry.

### Statistics

We used the Mann–Whitney *U*-test or Wilcoxon matched-pairs signed rank test to compare groups, and the Spearman’s rank test for correlations. Optimal cut-off points for the studied variables were determined with the Maximally Selected Rank Statistics package (Maxstat R-2.8.0, code available at: https://cran.r-project.org/web/packages/maxstat/index.html) [23]. Time-to-first-treatment (TTFT) was calculated between the date of diagnosis and the date of initial therapy, and TTFT curves compared by the Kaplan–Meier method; comparisons between groups were performed by the log-rank test. Cox regression was used to analyze the independent association of variables with TTFT. Statistical analyses were performed with SPSS software (version 15; SPSS Inc) and GraphPad (Prism 8; GraphPad Software). We considered two-sided *P* values < 0.05 to be statistically significant.

## Results

### Higher numbers of MDSCs and PMN-MDSCs circulate in the blood of CLL patients than of healthy controls

**Fig. 1:**
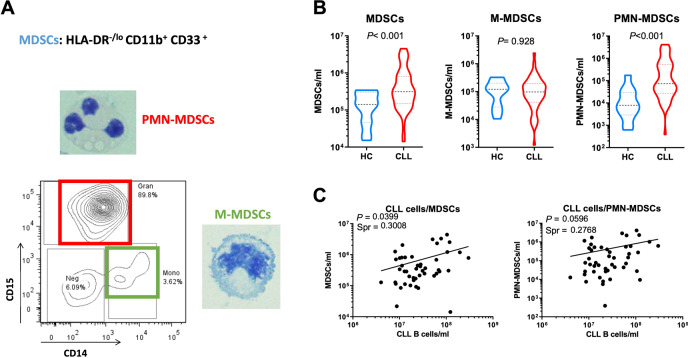
CLL PBMCs contain increased numbers of MDSCs and MDSC subsets. **A** Representative CD15/CD14 flow cytometry contour plot of MDSCs defined as HLA-DR^−/lo^CD11B^+^CD33^+^ cells (see Fig. S1A) and light microscopy images of cytospin preparations of FACS-sorted, Kwik-Diff ^TM^-stained CD15^+^ (PMN-MDSCs) and CD14^+^ (M-MDSCs) fractions from a CLL patient. **B** Violin plots of the absolute counts of circulating MDSCs, M-MDSCs, and PMN-MDSCs from 55 CLL patients and 12 gender- and age-matched healthy controls (HC); discontinuous lines correspond to median, 25th percentile and 75th percentile. Evaluated with Mann–Whitney test. **C** Correlation of absolute numbers of MDSCs and PMN-MDSCs with CLL B-cell counts. Evaluated with Spearman correlation test.

Using an HLA-DR^lo^CD11b^+^CD33^+^ phenotype to define MDSCs (Fig. S1) and expression of CD15 and CD14 to divide these into PMN-MDSCs and M-MDSCs subtypes (Fig. 1A and [24]), we found that the absolute numbers of MDSCs were significantly higher in 55 untreated CLL patients than 12 age-matched HCs (Fig. 1B). This difference was due to higher numbers of PMN-MDSCs, not M-MDSCs (Fig. 1B). Moreover, there was a significant direct correlation of the absolute numbers of MDSCs with the numbers of CLL B cells in the blood (Fig. 1C); the numbers of PMN-MDSCs trended with CLL B-cell counts, although this comparison was not significant (Fig. 1C).

### MDSC numbers correlate strongly with patient outcome

**Fig. 2:**
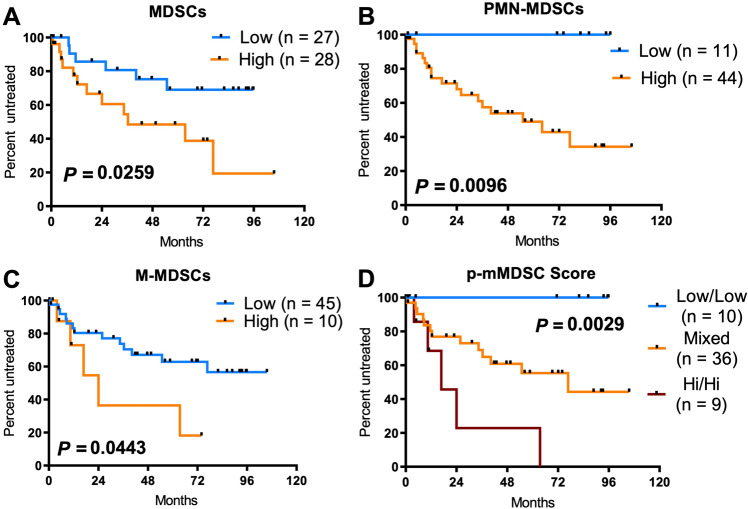
MDSC numbers associate with CLL patient time-to-first-treatment (TTFT). **A**–**C** Fifty-five CLL patients were dichotomized into High and Low count subsets based on the numbers of MDSCs, PMN-MDSCs, and M-MDSCs using the Maxstat package for R-2.8.0. TTFT curves were calculated by the Kaplan–Meier method and comparison between groups was performed by the log-rank test. **D** Patients were classified into three groups according to PMN-MDSC and M-MDSC values: low PMN-MDSCs and low M-MDSCs (Low/Low), high PMN-MDSCs and high M-MDSCs (Hi/Hi), and cases not fitting in either of the former categories (mixed).

Next, we divided patients into categories based on high and low MDSC, M-MDSC and PMN-MDSC counts using Maximally Selected Rank Statistics (see Methods) (Fig. S2) and assessed the categories for differences in clinical outcome. This indicated that patients with higher numbers of MDSCs or each subtype experienced significantly shorter TTFT (Fig. 2A–C), although there was a much stronger correlation with short TTFT for PMN-MDSCs than M-MDSCs.

Moreover, when patients were clustered by high or low M-MDSC and PMN-MDSC counts into High (Hi/Hi), Low (Low/Low) and Mixed (High/Low or Low/High) subgroups, respectively, (“p-mMDSC Score”), dramatic differences in clinical courses were found (Fig. 2D). The median TTFT for the High subgroup was significantly shorter than those of the Mixed and Low subgroups. Additionally, none of the patients in the Low subgroup required therapy, whereas all patients in the High and Mixed subgroups did.

Finally, using the Cox regression backward method, we performed a multivariate analysis by including the p-mMDSC Score and a series of other relevant prognostic indicators that significantly associated with TTFT in this cohort (Rai stage; IGHV mutation status; intracellular ZAP70 levels; and high-risk cytogenetics). Notably, only high-risk cytogenetics (11q^−^ and 17p^−^), ZAP70 levels, and the p-mMDSC Score retained significance (Table S2), documenting that the CLL B-cell extrinsic p-mMDSC Score influenced clinical course.

### Circulating PMN-MDSCs more effectively suppress autologous T-lymphocyte proliferation than M-MDSCs

**Fig. 3:**
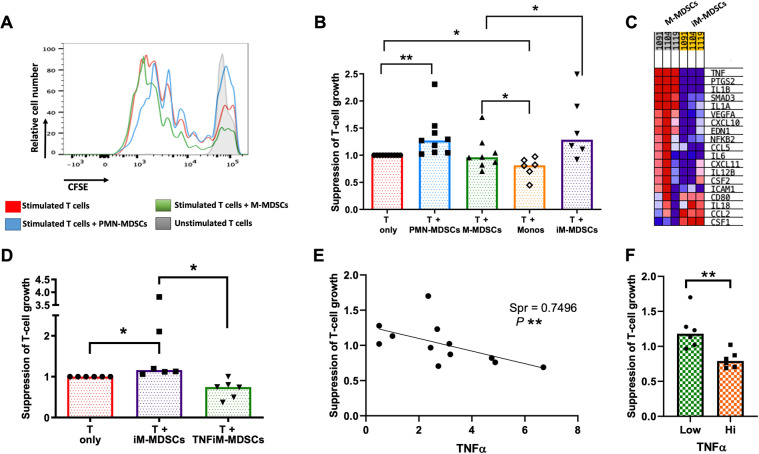
PMN-MDSCs more efficiently inhibit autologous T-cell proliferation in vitro than M-MDSCs, which are compromised by the action of TNFα. **A** Representative histogram illustrating CFSE dilution as a measure of the proliferation of T cells cultured alone or with autologous PMN-MDSCs and M-MDSCs upon activation by anti-CD3/CD28 beads plus IL2 for 72 h. **B** Proliferation of T cells stimulated alone (T only, *n* = 9) and co-cultured with FACS-sorted PMN-MDSCs, M-MDSCs, monocytes and in vitro induced iM-MDSCs (1:2 myeloid cell to T-cell ratio). Evaluated with Wilcoxon matched-pairs signed rank test. **C** Heat map of the expression levels of the genes identified by GSEA for hallmark TNFA signaling via NFKB, obtained after analyzing the gene expression of M-MDSCs and iM-MDSCs from three paired samples by real time PCR in a set of 92 immune-related genes (see Supplementary Information for methodological details). **D** Relative T-cell proliferation assessing the effect of adding TNF-α during M-MDSCs induction (TNFiM-MDSCs) in an independent experiment using the same six samples evaluated in section B. Evaluated with Wilcoxon matched-pairs signed rank test. **E** Correlation of TNFα serum levels vs. relative T-cell proliferation activity in the presence of M-MDSCs (*n* = 12). Evaluated with Spearman correlation test. **F** Impact on T-cell proliferation of serum TNFα levels in patients whose M-MDSCs inhibit or not vs. those that enhanced T-cell proliferation. Evaluated with Mann–Whitney test. *P* value: * <0.05; ** <0.01; *** <0.001.

To evaluate the functional capacities of CLL MDSCs, we FACS-isolated patient M-MDSCs and PMN-MDSCs and tested their abilities to inhibit autologous T-cell proliferation (Fig. 3A). Only PMN-MDSCs consistently significantly suppressed dividing T cells (Fig. 3A, B); the effects of M-MDSCs varied and were insignificant. In contrast, autologous monocytes significantly enhanced T-cell expansion, when compared with the proliferation of T cells alone or T cells plus PMN-MDSCs or M-MDSCs (Fig. 3B). Notably, although PMN-MDSCs exhibited higher surface expression of proteins that promote contact with T cells (CD124 and CD80), they also over-expressed proteins that block the functional consequences of T-cell contact, i.e., checkpoint molecules PD-L1/2 (CD273 and 274). Combined, these actions likely reduced T-cell effector function. In addition, PMN-MDSCs expressed higher levels of S100A9, which promotes inflammatory reactions that can lead to the production of reactive oxygen species and related molecules such as iNOS and indoleamine (IDO), which were higher in these cells and have been associated with the inhibition of T-cell function [25]. M-MDSC expressed TGFβ membrane protein (Fig. S3), consistent with M-MDSCs being less immunosuppressive than PMN-MDSCs.

### The inability of circulating M-MDSCs to suppress autologous T-lymphocyte proliferation is an acquired deficiency due to the action of TNFα

To determine if CLL M-MDSCs were inherently defective in T-cell suppression activity or if they developed the deficiency due to external influences, we induced M-MDSCs (iM-MDSCs) by stimulating CD33^+^ cells from individual CLL patients with GM-CSF, IL10, and IL-6 [21]. iM-MDSCs significantly suppressed T-cell proliferation (Fig. 3B), and the magnitude of suppression was virtually identical to that of PMN-MDSCs taken ex vivo (Fig. 3B). Hence, the inability of M-MDSCs to suppress in CLL was induced and not intrinsic.

In order to define transcriptomic changes caused by microenvironmental input in CLL M-MDSCs, we analyzed gene expression in M-MDSCs and iM-MDSCs from the same patients and compared the findings by GSEA. This analysis identified the HALLMARK TNFA SIGNALING VIA NFKB gene set as most similar (Figs. 3C, S4), as we had seen when measuring membrane protein (Fig. S3).

We next sought to ascertain the effects of TNFα on the T-cell suppressive activity of iM-MDSCs. Indeed, addition of TNFα to the iM-MDSC-inducing cocktail led to a significant reduction in the ability of the iM-MDSCs to suppress T-cell proliferation (Fig. 3D). This indicated a direct relationship between the action of TNFα and M-MDSC function.

Finally, we identified a series of CLL patients with divergent levels of serum TNFα (*n* = 12) and compared the abilities of M-MDSCs from these to suppress autologous T-cell growth in vitro. This revealed a significant direct, linear relationship between high TNFα levels and impaired suppression (Fig. 3E, F).

Thus, TNFα is involved in the diminished capacity of M-MDSCs to suppress autologous T-cell growth in CLL, and the circulating level of TNFα directly correlates with the degree of T-cell immunosuppression in individual patients. Thus, although elevated TNFα is viewed as a negative prognostic indicator in CLL [26, 27], high TNF levels can be somewhat beneficial by diminishing MDSC-induced T-cell suppression.

### Correlation of MDSC subtypes with the levels of T-helper subsets in patients in vivo

Since mature T cells fall into distinct functional subsets that have discrete influences on the development and progression of cancer [28, 29], we next studied if CLL-derived MDSCs influenced the relative distribution of Th subsets, thereby affecting CLL-cell growth and influencing clinical outcome. Figure S5A describes the flow cytometry gating strategy for these studies.

These analyses indicated that CLL patients have significantly higher numbers of circulating CD4^+^ and CD8^+^ cells than age-matched HCs (Fig. S6A). Additionally, within the CD4-cell compartment there were more Th1 (IFN-γ^+^), Th2 (IL-4^+^), and T regulatory (Treg) (FoxP3^+^) cells in CLL patients than in HCs (Fig. S6B), consistent with the documented memory T-cell expansion in CLL [9]. Although the level of Th17 cells was greater in CLL, this did not reach statistical significance.

Next, we correlated the numbers of T-cell subsets and the various CD4^+^ subpopulations with MDSC counts in a subgroup (*n* = 18) of the patients studied (Table 1, Fig. S7). Although the size of the total MDSC population correlated directly with both CD4^+^ and CD8^+^ T cells (Fig. S7A, B), M-MDSCs associated significantly with CD4^+^ cells (Fig. S7D) and PMN-MDSCs with CD8^+^ cells and CD4^+^ Tregs (Fig. S7E, F). Additionally, total MDSC numbers directly correlated with Th2 (Fig. S7G). Only M-MDSCs positively associated with Th1 cells (Fig. S7H), implying that PMN-MDSCs correlate more with Th2, albeit not significantly possibly due to the number of patients studied.

**Table 1:** Relation of MDSCs and their subtypes to T-cell subpopulations.

			In vivo				In vitro		
T-cell population/Th-cell subset	Timing	*N* (cases)	MDSC numbers	PMN-MDSC numbers	M-MDSC numbers	*N* (cases)	PMN-MDSC	M-MDSC	Monocytes
	Before ibrutinib Rx								
CD3^+^		55	↑↑	–	↑↑	20	NA	NA	NA
CD4^+^		55	↑↑↑	–	↑↑↑	20	NA	NA	NA
CD8^+^		55	↑↑↑	↑↑	–	20	NA	NA	NA
Tregs		27	↑↑↑	↑	–	20	–	↓↓	↓↓↓
Th1		18	–	–	↑	20	–	–	↑
Th2		18	↑	–	–	20	–	–	↑
Th17A		18	–	–	–	20	–	–	–
Th17F		18	–	–	–	20	↑	–	–
	During ibrutinib Rx								
CD3^+^		16	–	–	↑	8	NA	NA	NA
CD4^+^		16	–	↓	↑	8	NA	NA	NA
CD8^+^		16	–	–	↑	8	NA	NA	NA
Tregs		16	–	↓	–	8	↑	↑	↑
Th1		16	–	–	–	8	↑	↑	↑↑
Th2		16	–	↓	↑	8	–	–	–
Th17A		16	–	↓	–	8	–	–	–
Th17F		16	–	–	↑	8	↑	–	–

Direction of arrow indicates a significant positive or negative correlation (in vivo) and increase or decrease over control (in vitro); the number of arrows indicates the degree of statistical significance (1 arrow <0.05, 2 arrows <0.01, and 3 arrows <0.001) compared to control.

*NA* Not applicable.

### Influence of MDSCs on T-helper cell subset polarization in vitro

**Fig. 4:**
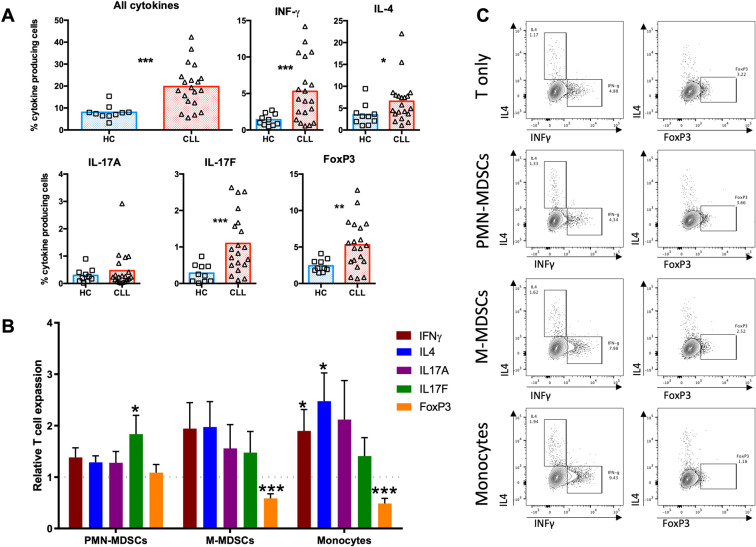
MDSCs and monocytes alter naive CD4^+^ T-cell differentiation in a subset-biased manner. **A** FACS-sorted naive CD4^+^ T cells (T_N_s; CD3^+^CD4^+^CD45RO^−^CD62L^+^) from 20 CLL patients and ten HC were stimulated with anti-CD3/CD28 beads plus IL2 in vitro for 6 days and the percentages of cytokine-producing (IFN-γ, IL4, IL17A, and IL17F) and FoxP3-expressing cells were evaluated by flow cytometry. Evaluated with Mann–Whitney test. **B** Relative fold change of T-cell subset differentiation upon stimulation with anti-CD3/28 beads plus IL-2, comparing FACS-sorted T_N_s alone (dotted line) or with PMN-MDSCs, M-MDSCs, and monocytes (1:2 myeloid cell to T-cell ratio). Evaluated with Wilcoxon matched-pairs signed rank test. **C** Representative flow cytometry scatter plots of T cells after culture with the indicated condition. *P* values: * <0.05; ** <0.01; *** <0.001.

To develop a mechanistic relationship between MDSCs and Th subpopulations, we explored the effects of PMN-MDSCs and M-MDSCs on autologous T_N_-cell differentiation in vitro (Fig. S5B). First, CLL T_N_s were stimulated with anti-CD3/28 beads plus IL-2 in the absence of other cells. CLL T cells generated a significantly higher percentage of cytokine-producing cells than T_N_ cells from HCs (Fig. 4A), implying a fundamental difference between T_N_ cells in CLL versus HCs.

Next, CD4^+^ CLL T_N_ cells were stimulated in the same manner in the presence of the two MDSC subtypes. PMN-MDSCs led to a significant, selective expansion of IL-17F-producing cells compared to results obtained when T_N_ cells were stimulated alone (Fig. 4B, dotted line). Conversely, upon co-culturing CLL T_N_s with M-MDSCs or monocytes, each led to significant reductions in Tregs and monocytes resulted in significant increases in Th1 and Th2 cells (Fig. 4B).

### Ibrutinib therapy selectively alters the numbers of MDSCs, and CD4^+^ and CD8^+^ T-cells and Th-cell subsets in vivo

**Fig. 5:**
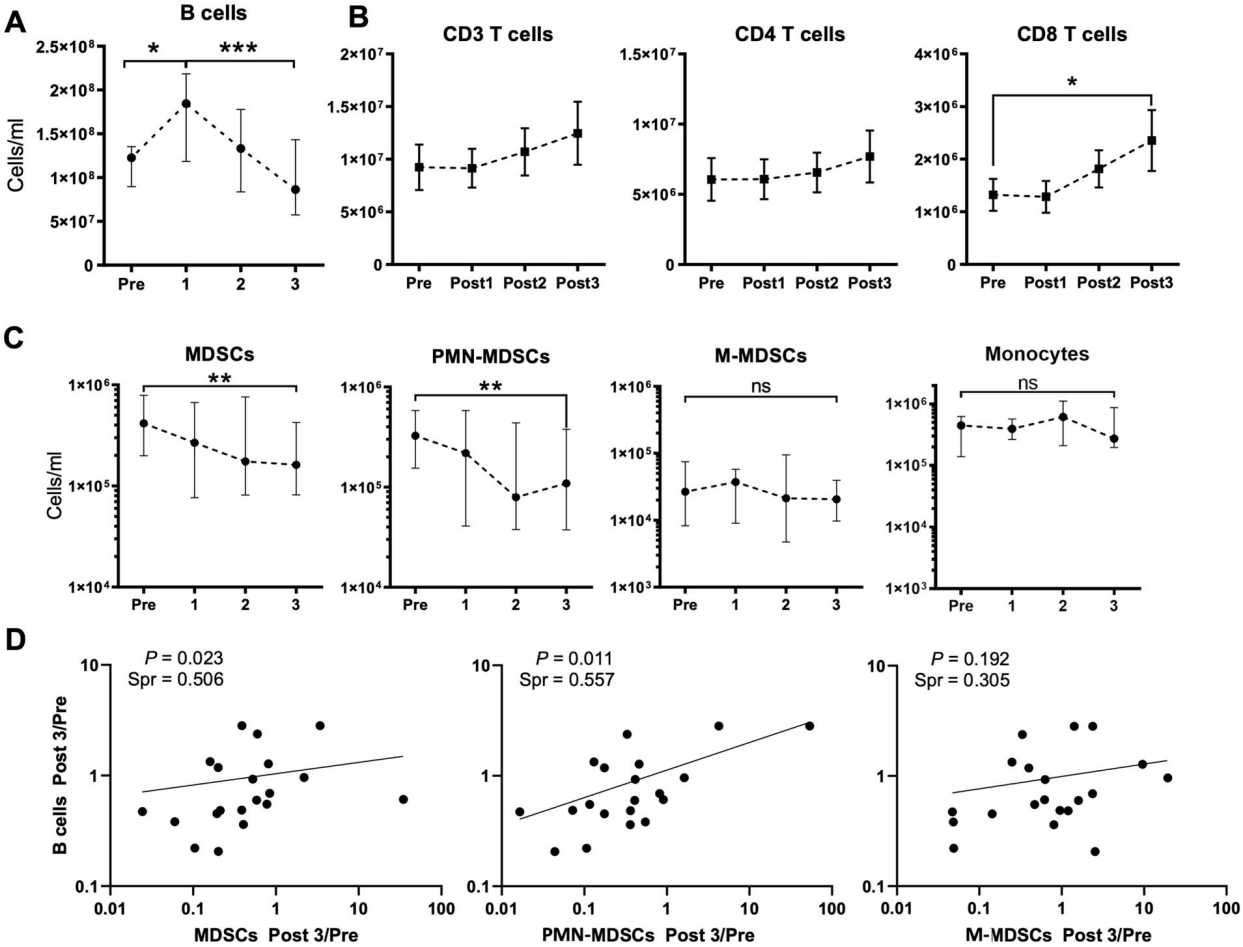
Ibrutinib treatment alters the numbers and activation states of MDSCs in vivo. Absolute counts of B cells (**A**), T cells (CD3^+^, CD3^+^ CD4^+^, and CD3^+^ CD8^+^) (**B**), and MDSCs and subsets (**C**) in 20 patients prior to initiating (Pre) and after 1, 2, and 3 months on ibrutinib treatment. Statistics reflect comparisons between pretreatment and the different time points for a specific population. Dots correspond to median and error lines correspond to interquartile range and discontinued lines to the connection of the median values and difference were evaluated with Wilcoxon matched-pairs signed rank test. **D** Correlation between the ratio of CLL B cells and MDSCs at 3rd month vs. prior to initiating treatment, evaluated with Spearman correlation test. *P* values: * <0.05; ** <0.01; ***<0.001.

Using cryopreserved PBMCs from a cohort of previously untreated patients who received ibrutinib as initial single agent therapy [30], we analyzed MDSCs and the MDSC subtypes before and the first 1, 2, and 3 months on treatment. As expected [31], 1 month after initiating ibrutinib therapy, CD5^+^ B-cell counts increased significantly and then progressively fell over time (Fig. 5A). Correspondingly, although total T-cell and CD4^+^ and CD8^+^ T-cell counts increased progressively (Fig. 5B), only CD8^+^cells reached a level significantly greater at month 3. In contrast, over the same period, ibrutinib therapy led to a progressive fall in the numbers of MDSC and PMN-MDSC that achieved significance by the 3rd month. M-MDSC and monocyte numbers did not change with therapy (Fig. 5C).

The in vivo changes in MDSCs and in T-cell numbers post ibrutinib therapy led to altered relationships between MDSCs and T lymphocytes. Specifically, whereas PMN-MDSCs did not associate significantly with CD4^+^ T-cell numbers before therapy, they correlated negatively with CD4^+^ cells afterwards. Conversely, M-MDSCs, which did not associate significantly with CD8^+^ T cells numbers before therapy, correlated positively with CD8s afterwards (Table 1, Fig. S8).

In line with this preferential decrease in PMN-MDSC and not M-MDSC numbers, the relative change in CLL B-cell levels before and after therapy correlated significantly with the changes in MDSC and PMN-MDSC levels pre- and post-treatment (Fig. 5D). This was not the case for M-MDSCs (Fig. 5D). Thus, the beneficial effects of ibrutinib only correlate with the fall in leukemic B cells and PMN-MDSCs.

Concomitant with these changes in MDSCs, CD4^+^ and CD8^+^ T-cells, and leukemic B-cell numbers and ratios, a significantly higher level of the Th1 (IFN-γ^+^) subset was reached at month 2, and this persisted through the 3rd month (Fig. S9). Likewise, Treg (FoxP3^+^) cells increased progressively over the 3-month period, although this did not achieve statistical significance. Finally, Th17F (IL-17F^+^) cells were transiently increased at month 2 but regressed to pre-therapy levels by month 3.

### Ibrutinib selectively alters MDSC effects on T-helper subsets in vitro

**Fig. 6:**
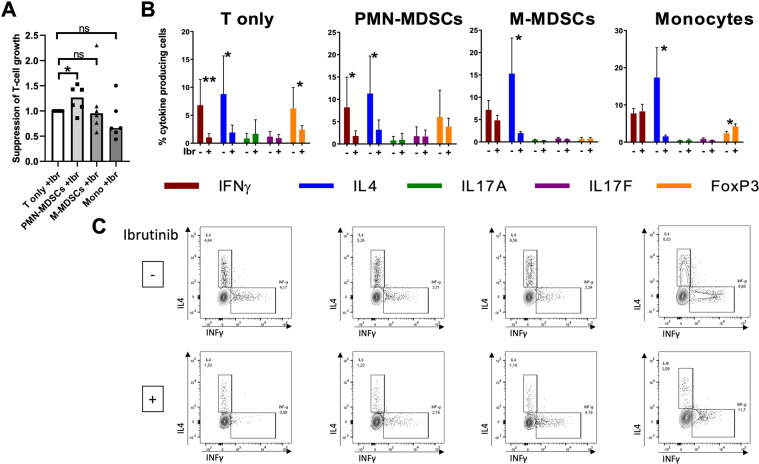
Ibrutinib does not affect MDSC T-cell suppressive function, but does alter induction of Th subsets from naive T-cells in vitro. **A** Relative T-cell proliferation from six patients evaluated by dilution of CFSE in T cells alone activated with anti-CD3/CD28 beads plus IL2 (set as 100% T-cell proliferation) and co-cultured with FACS-sorted PMN-MDSCs, M-MDSCs, and normal monocyte in the presence of 1ug/ml of ibrutinib for 72 h. **B** Percentage of cytokine-producing cells evaluated by flow cytometry of FACS-sorted T_N_ cells after 6 days of stimulation in the absence or presence of ibrutinib (1 µg/ml) without and with the co-culture with PMN-MDSCs, M-MDSCs and normal monocytes (*n* = 8). Bars represent the median and the interquartile range. Difference valuated with Wilcoxon matched-pairs signed rank test. **C** Representative flow cytometry scatter plots of T cells after culture with the indicated condition. *P* values: * <0.05; ** <0.01; *** <0.001.

Next, we explored the effects of in vitro exposure to ibrutinib on PMN-MDSC and M-MDSC function. First, we asked if ibrutinib altered the effects of PMN-MDSCs and M-MDSCs and monocytes on autologous T-cell proliferation. Notably, this was not the case since the drug did not significantly modify the direct effects of either subtype on T-cell growth in vitro: PMN-MDSCs still reduced proliferation and M-MDSCs continued to have variable and insignificant effects (Fig. 6A).

This fact allowed us to carry out co-cultures of T_N_ cells with MDSCs in the presence of ibrutinib under conditions that would occur in patients and to compare the effects to T cells cultured alone. Although the drug selectively and significantly reduced in vitro maturation of T_N_s to Th1 (IFN-γ^+^), Th2 (IL-4^+^), and Treg (FoxP3^+^) cells (Fig. 6B), when T_N_ cells were cultured with MDSC subtypes or monocytes in the presence or absence of ibrutinib, different patterns of response were noted (Fig. 6B).

In PMN-MDSC-containing cultures, ibrutinib led to significant falls in the generation of IFN-γ- and IL-4-producing cells, without a significant change in Tregs. In M-MDSC-containing cultures, ibrutinib resulted in a selective decrease in maturation to Th2/IL-4^+^ T cells without affecting Th1 or Treg levels. Additionally, in monocyte-supplemented cultures, exposure to ibrutinib significantly dropped differentiation to IL-4-producing cells and enhanced the maturation to Tregs. Finally, only PMN-MDSCs reduced the numbers of Th1 cells. In vitro maintenance of Th1s by M-MDSCs and monocytes (Fig. 6B) is consistent with the shift in the balance of T-cell subsets toward the immunostimulation observed at 3 months of ibrutinib treatment in vivo (Fig. 5A).

## Discussion

Here we have quantified the absolute numbers of MDSCs and their lineage-based subtypes in the blood of CLL patients, their effects on the growth and maturation of naive T-lymphocytes to Th subsets, and their association with patient outcome. We have also explored the impact of ibrutinib treatment on these parameters. Our studies indicate greater numbers of MDSCs in CLL patients than HCs. MDSC numbers correlate with Th2 and Treg cells (Table 1), consistent with an association with the immunosuppressive property of MDSCs [8–10, 12, 13]. Notably, significant differences in cell associations and functional capacities were found when MDSCs were divided into their subtypes, a finding not previously reported in CLL.

PMN-MDSCs represented the major of MDSCs component in the blood of CLL patients (Fig. 1), and their numbers correlated significantly with CD8^+^ cells (Fig. S6E), Tregs, and Th17 cells in vivo (Table 1, Fig. S6). Moreover, they consistently reduced autologous T-cell proliferation in vitro (Fig. 3A, B) and induced higher levels of IL-17F^+^ T cells in co-cultures with T_N_s (Fig. 4B), resembling the in vivo situation (Table 1). Collectively, these findings suggest a prominent role for PMN-MDSCs in the immune dampening capacities of MDSCs in CLL.

In contrast, M-MDSCs were not numerically different between CLL and HC. This differs from that reported by others [16–18], possibly due to our quantification of absolute numbers rather than percentages of M-MDSCs and our flow cytometry strategy defining MDSCs. However, the most striking difference between M-MDSCs and PMN-MDSCs was the inability of the former to suppress autologous T-cell division (Fig. 3A, B). Since this observation also differed from that of another [17], we investigated M-MDSC-mediated T-cell immunosuppression more deeply, asking if the defect was primary or secondary. CLL M-MDSCs induced in vitro (iM-MDSCs) could significantly impair T-cell proliferation (Fig. 3B), indicating that the inability to do so in vivo was not inherent but acquired, presumably due to influences from the TME [13, 15]. Mechanistically, this acquired deficiency was a consequence of TNFα, since in vitro induction of suppressive M-MDSCs was blocked by TNFα, and serum TNFα levels in patients directly correlated with T-cell suppression function (Fig. 3D, E). Further exploration is needed to fully understand if additional molecules altered in CLL [32] are also involved in modifying M-MDSCS function.

Moreover, M-MDSCs differed from PMN-MDSCs by (1) higher expression of genes and proteins supporting an inflammatory response and lower expression of checkpoint molecules, (2) correlation with CD4^+^ (Fig. S6E) and Th1 (Table 1, Fig. S7) cells in vivo, and (3) significantly reducing maturation of autologous T_N_s to Tregs, similar to that observed with monocytes. Additionally, monocytes promoted Th1s and Th2s (Fig. 4B) similar to M-MDSCs, although this did not reach significance possibly due to the number of cases tested.

Our M-MDSC findings regarding Tregs in CLL differ from those of Jitschin et al. who reported that M-MDSCs drove Treg expansion and suppressed proliferation of CD2/CD3/CD28-stimulated T cells [17]. We believe this difference is due to experimental design. In the Jitschin et al. study, mMDCS were generated in vitro from monocytes taken from PBMCs of HCs after co-culture with CLL B cells. Similarly, the Tregs studied came from the same co-cultures. Hence both cell types—MDSCs/M-MDSCs and Tregs—came from normal, healthy individuals, not CLL patients as in our experiments. Additionally, for Jitschin et al. the MDSCs/M-MDSCs and Tregs were induced during an allogeneic co-culture. These data are informative, but they address the effects of CLL B cells on allogeneic normal T cells in vitro and might not reflect the in vivo situation. Since our data derive from cells from CLL patients generated in the natural setting, we believe our findings have physiologic and pathobiologic relevance.

Clinically, we found that MDSCs and each subtype correlated with inferior patient outcome (Fig. 2A–C). Although this observation is consistent with prior reports indicating that M-MDSCs can presage time to progression [16, 17], our data revealed that PMN-MDSCs had a stronger association with aggressive disease than mMDCS (Fig. 2A–C). In addition, stratifying cases based on the relative numbers of PMN-MDSCs and M-MDSCs (p-mMDSC Score) identified three sets of patients with very different TTFT. No patient in the Low p-mMDSC Score group and all patients in the High p-mMDSC Score group required therapy. Additionally, the p-mMDSC Score was an independent prognostic indicator in multivariate analysis (Fig. 2D, Table S2), confirming a pathologic role for PMN-MDSCs in CLL.

Collectively, our findings concur with the principle that Th1 responses, linked here with M-MDSCs and monocytes, preferentially promote anti-tumor immunity, and that Th2 responses, associated here with PMN-MDSCs, support cancer growth. Notably, the difference between PMN-MDSCs and M-MDSCs and these two distinct T-cell subset actions resemble those in autoimmune hepatitis that link MDSCs with Th1 cells causing immune-mediated liver injury [33] and in a mammary adenocarcinoma model that correlate Th2 cells with disease progression [34]. However, since M-MDSCs do associate with worse patient outcome (Fig. 2), this MDSC subtype must also have detrimental effects for patients that are mediated by undefined mechanisms. In this regard, cells with an M-MDSC phenotype but lacking T-cell suppressive activity can play a significant role in tumor development and disease progression in the setting of chronic inflammation [35, 36], where factors produced by Th1 cells support CLL-cell survival and proliferation in vitro as well as in vivo in mouse models [37, 38]. Moreover, M-MDSCs can suppress CD8-cell function by inducing tolerance, not necessarily by inhibiting CD8-cell proliferation [39]. Finally, M-MDSCs can also function as T cell-independent mediators of tumor proliferation as occurs in squamous cell carcinoma [40]. Since, as we report here, MDCSs can modulate the Th repertoire, they could be regulating B-cell numbers and functions directly and/or indirectly. In addition, this multidirectional relationship between MDSCs, T cells, leukemia cells, and the TME could be the basis for some of the discrepancies between the correlations observed in vivo and in vitro.

Inhibition of BTK function by ibrutinib has had a major impact on limiting CLL disease progression [41]. Hence, we analyzed changes in MDSC and T-cell subsets in CLL patients receiving this therapy, keeping in mind that the drug’s major target, BTK, is expressed by B cells and other cells (including MDSCs) [42–47], and that ibrutinib can inhibit other kinases in the TEC (e.g., ITK) and epidermal derived growth factor receptor families [48]. Unlike the numbers of B and T cells that transiently increased in the blood after ibrutinib therapy, neither MDSCs nor the subtypes increased upon therapy initiation. Rather, for total MDSCs and PMN-MDSCs, there was a progressive decline after 3 months of treatment that was not found for M-MDSCs nor monocytes (Fig. 5C), indicating a differential effect on the MDSC subtypes with M-MDSCs behaving more like the lineage-linked monocytes. This dichotomy implies that, upon initiation of ibrutinib therapy, MDCSs and PMN-MDSCs do not emigrate from tissue niches or they more rapidly return there from the blood. Importantly, the diminution in PMN-MDSC numbers correlated with the fall in CLL B-cell numbers. Since such a relationship was not found for M-MDSCs, one of the beneficial actions of the drug might be due to its effect on PMN-MDSCs. This could be indirect, relating to reductions of cytokines such as TGF-β, IL-6, or the immune suppressor and MDSC-inducer cytokine, IL-10 [49–51], all of which are over-expressed in CLL [32]. Regardless, ibrutinib reduced the numbers of tumor-promoting PMN-MDSCs without altering the levels of immunostimulatory M-MDSCs and monocytes.

In a related manner, ibrutinib treatment led to a significant increase in IFN-γ-producing Th1 cells at the 3-month time point (Fig. S9), along with a trend toward higher Tregs. Moreover, when we cultured T_N_ cells with ibrutinib in the absence of PMN-MDSCs, M-MDSCs, or monocytes, an increase in Th1, Th2, and Tregs was observed, Th phenotypes that combines immunostimulatory (Th1) and immunosuppressive (Th2 and Tregs) features. However, the addition of PMN-MDSCs, M-MDSCs, or monocytes to T-cell cultures led to a fall in IL-4^+^ Th2 cells for each. Conversely, only the addition of PMN-MDSCs concomitantly led to a fall in Th1 cells (Table 1, Fig. 6B). Thus, M-MDSCs and monocytes eliminated the immunosuppressive Th2 phenotype but preserved the Th1 immunostimulatory phenotype, whereas PMN-MDSCs removed both. Monocytes also promoted the generation of more Tregs.

Of note, mice in which IL-2-inducible T-cell kinase (ITK), an enzyme in the same family as BTK, has been genetically eliminated develop a preferential expansion of Th1 cells [52] and eventually acquire greater numbers of Tregs over Th17s [53]. Since ITK is absent in these animals from birth, the selective development of Th1 and Tregs most likely occurs through T_N_-cell maturation and not by selective expansion of existing T-cell subsets. Of note, the co-culture of CLL T_N_ cells with M-MDSCs or monocytes in the presence of ibrutinib led to the same T-cell subsets found after genetic removal of ITK, which, as mentioned, is also a target of ibrutinib. Similarly, in vitro studies using CLL PBMCs indicate that ibrutinib favors Th1 cell expansion [54]. Since CLL patients have an expansion of memory T cells and a relative paucity of T_N_s [9], the Th1 increase more likely results from Th1 expansion.

Hence, a part of ibrutinib’s beneficial effects could come about by its actions on MDSCs and their subtypes. Consistent with this, ibrutinib can inhibit iM-MDSCs generation in vitro [47], thereby augmenting the action of TNFα in preventing T-cell suppression, and can promote the maturation of M-MDSCs to dendritic cells [55]. Together, these immunostimulatory actions could delay clinical progression by preventing or obviating M-MDSC-mediated T-cell suppression and increasing anti-tumor response.

Despite these actions on the Th compartment, ibrutinib did not alter the differential abilities of PMN-MDSCs and M-MDSCs to suppress T-cell growth (Fig. 6A). In this regard, cryopreservation can affect MDSC function [56]. Since in our studies we needed to test and compare samples from multiple patients simultaneously, we used only cryopreserved samples. Therefore, we compared the T-cell suppressive actions of PMN-MDSCs, M-MDSCs, and monocytes from the same patient sample, before and after cryopreservation. Freezing and thawing only affected the downregulatory actions of PMN-MDSCs (Fig. S10). Hence, the differences in T-cell suppression we identified between PMN-MDSCs and M-MDSCs would likely be greater if fresh samples were used.

Finally, since MDSCs are involved in creating/maintaining an immunosuppressive environment, preferentially lowering or inactivating their numbers or actions would likely be beneficial for patients. PMN-MDSCs appear to be the preferred subtype to target, since they more effectively induce immune suppression in CLL. Similarly, since M-MDSCs appear to be less disease-promoting and more immunostimulatory than PMN-MDSCs, strategies to selectively enhance these M-MDSC actions might also be therapeutically valuable.

## Supplementary information

Supplementary Material
