# Supplementary material for: Myeloid-derived suppressor cell subtypes differentially influence T-cell function, T-helper subset differentiation, and clinical course in CLL
Source: Leukemia. 2021 May 2;35(11):3163–75. doi: 10.1038/s41375-021-01249-7 (PMC8550941; doi:10.1038/s41375-021-01249-7)
Supplement: Supplementary file 1 — Supplementary Material [file 41375_2021_1249_MOESM1_ESM.pdf]

Supplementary Materials and Methods

Flow cytometry and FACS sorting. The frequency and phenotype of MDSCs as well as other populations were evaluated by flow cytometry (FC) using a BD LSR II (BD Immunocytometry Systems). First, we labelled dead cells using the LIVE/DEAD™ Fixable Violet Dead Cell Stain Kit or the Far Red Dead Cell Stain Kit Life Technology), and then, one million cells were exposed to mAbs reactive with various surface markers (Table S3). When required, samples were fixed and permeabilized using the Cytofix/Cytoperm kit (BD Biosciences) and stained according to manufacturer's recommendations. However, for FoxP3 and listed cytokines, the Human FoxP3 Buffer Set (BD Biosciences) was used. FC data were analyzed by FlowJo V10 software (TreeStar Inc.).

FACS Aria II (BD Immunocytometry Systems) was used for cell sorting. Due to the high numbers of CLL B cells in relation to MDSCs and T lymphocytes, some samples were pre-enriched for non-B cells by magnetic removal of $CD19^{+}$ cells using the EasySep™ Human CD19 Positive Selection Kit (StemCell Technology).

Cell morphology defined by light microscopy. In some instances after FACS-sorting, an aliquot was evaluated for cell morphology by light microscopy. Between 5-30 x 10^{3} cells were transferred to a slide using a Cytospin 2 centrifuge (Shandon). After drying, cells were fixed and stained with the Kwik-Diff™ Staining Kit (ThermoFisher). Microscopy was performed using an Olympus BX40 fitted with UplanFl 10x/0.30, 20x/0.50, 40x/0.65, and 60x/1.25 objectives.

Quantitative RT-PCR for MDSC gene expression. Expression of 92 genes in FACS-isolated mMDSCs and i-mMDSCs was determined by RT-PCR using the TaqMan® Array Human Immune Response 96-well Plate (Applied Biosystems). RNA was isolated from both cell populations using the NucleoSpin® RNA kit (Macherey-Nagel), and single-stranded cDNA was generated using SuperScript® II Reverse Transcriptase (Thermos). Gene expression was carried-out by amplifying mRNA in an Applied Biosystems 7900HT Fast Real-Time PCR System and analyzed using the SDS 2.4 and RQ Manager 1.2.1. Results were calculated using the method $2^{-\Delta\Delta}$ and using *18S*, *GAPDH*, *HPRT1* and *GUSB* as endogenous controls.

Gene set enrichment analysis (GSEA). To gain insight into the molecular pathways differentially expressed between mMDSCs and i-mMDSCs, GSEA was performed using version 3.0 software. Gene sets used for the enrichment analysis were downloaded from the Molecular Signatures Database (MsigDB, h.all.v6.2.symbols.gmt). The gene sets with false discovery rates < 0.25 were considered significantly enriched.

Multiplex assay of secreted cytokines. Soluble levels of IFN-γ, TNF-α, IL-4, IL-13, IL-17A, IL-17F, and IL-10 were measured using MSD U-PLEX Platform (MSD) following the manufacturer's instructions. Briefly, each primary antibody was coupled to a unique linker to identify its specificity, and then all were mixed and the assay plate coated overnight. After washing, plates were loaded for 1 hour with calibrators and samples, washed, and then incubated for 1 hour with the detection antibody. Finally, after washing, the plate was read in a SECTOR S 600 (MSD). Analysis was performed using DISCOVERY WORKBENCH 4.0 (MSD).

Supplementary Tables

Table S1: Patient clinicobiological characteristics

	CLL patients (n = 55)	CLL patients treated with ibrutinib (n = 20)
**Age, years**	59	64
**Gender (% M:F)**	65	60
**Rai stage, n**		
**0**	30	0
**1**	12	4
**2**	6	5
**3**	5	8
**WBC (10^6 cells/ml)**	32 [15-134]	130 [40-270]
**Unmutated IGHV (%)**	25 (50)	11 (55)
**ZAP70 ≥ 20% (%)**	15 (36)	10 (50)
**CD38 ≥ 30% (%)**	12 (27)	5 (25)
**High-risk cytogenetics[a] (%)**	11 (26)	11 (55)
**Follow-up (months)**	81 [0.2-108]	na

[a]: 17p- or 11q-
na: not applicable

Table S2: Cox regression backward multivariate analysis. Analysis eliminates one factor in each iterative round until independent factors are identified.

		P value	Hazard Ratio (95% CI)
Step 1	IGHV mutation status	0.153	0.268 (0.044 - 1.633)
	ZAP70 levels^	0.127	3.692 (0.691 - 19.742)
	High-risk cytogenetics*	0.002	7.357 (2.037 - 26.57)
	RAI stage	0.256	2.588 (0.501 - 13.37)
	p-mMDSC Score	0.061	9.783 (0.896 - 106.772)
Step 2	IGHV mutation status	0.148	0.267 (0.045 - 1.597)
	ZAP70 levels^	0.096	4.106 (0.778 - 21.669)
	High-risk genetics*	0.002	7.845 (2.145 - 28.687)
	p-mMDSC Score	0.053	10.212 (0.966 - 107.942)
Step 3	ZAP70 levels^	0.006	7.936 (1.799 - 35.005)
	High-risk genetics*	0.001	9.263 (2.502 - 34.293)
	p-mMDSC Score	0.039	12.174 (1.135 - 130.632)

^: 20% cutoff for high and low ZAP70 levels *

*: High-risk genetics = 17p- or 11q-

Table S3: Antibody list

Antigen	Color	Company	Ref. number
CD3	A700	BioLegend	317340
CD3	PE-Cy7	eBioscience	25-0038-42
CD4	V450	BD	560345
CD8	V500	BD	561617
CD11b	PE-Cy7	BD Bioscience	557743
HLA-DR	APC	BD Bioscience	559866
CD33	V450	BD Bioscience	561157
CD14	FITC	BD Bioscience	347493
CD15	PE	BD Bioscience	562371
CD19	eFluor450	eBioscience	48-0193-82
IFN-g	A488	eBioscience	53-7319-42
IL-4	APC	BD Bioscience	561233
IL-17A	PERCP	BD	560799
IL-17F	PE	eBioscience	12-7169-41
FoxP3	A488	BD Bioscience	560047
CD5	PerCP-cy5.5	BD	341089
CD45RO	APC	BD	340438
CD62L	PE	eBioscience	12-0629-42

Supplementary Figures

Figure S1: Flow cytometry marker and gating strategies to identify MDSCs

Flow cytometry strategy for the identification of MDSCs and MDSCs subsets.

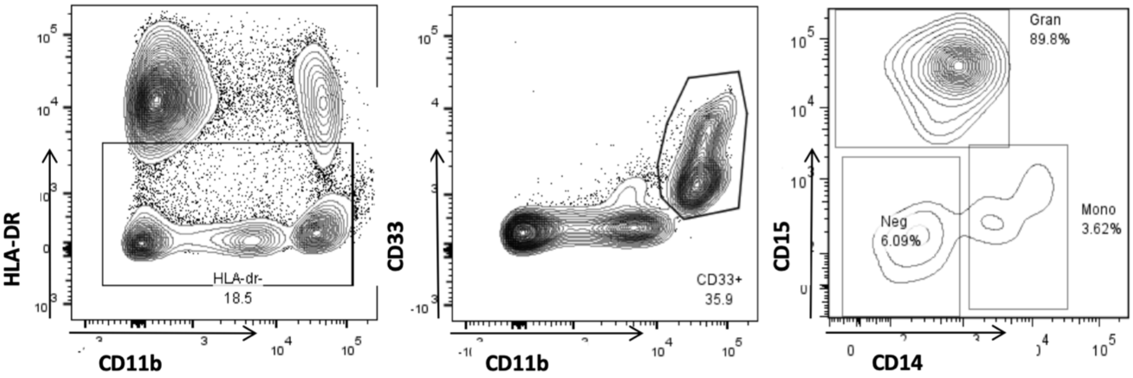

Figure S2: Cut off points for the Studied Variables

Optimal cut-off calculated for the studied variables was determined with the Maximally Selected Rank Statistics (Maxstat) package for R-2.8.0 and indicated with a dotted line.

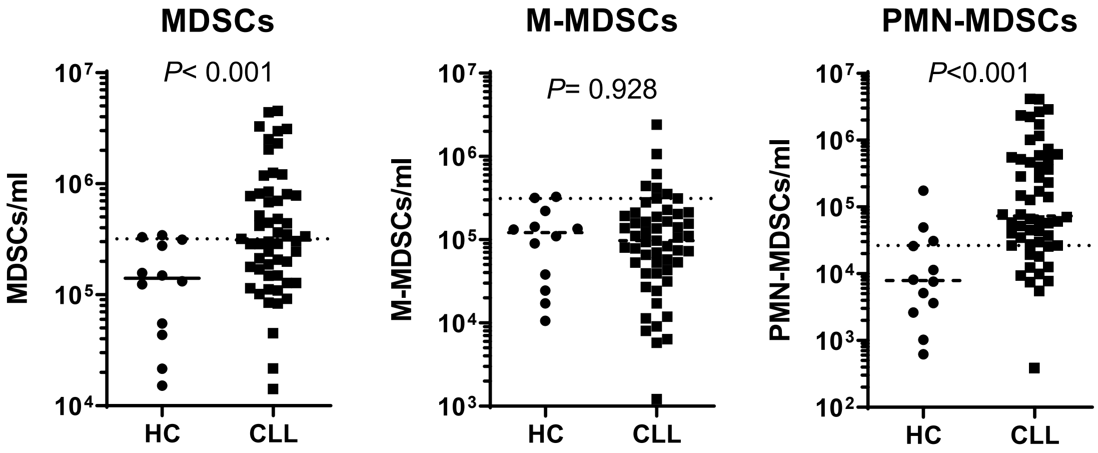

Figure S3: Differences between PMN-MDSCs and M-MDSCs

A. Differentially expressed genes (p<0.05) evaluated by qPCR, between 3 pair of PMN-MDSCs and M-MDSCs. **B.** Signal obtained by flow cytometry for indicated molecules on/in M-MDSCs and PMN-MDSCs in samples from 36 CLL patients. Black arrows indicate a significantly higher expression in PMN-MDSCs vs M-MDSCs; gray arrows indicate the opposite comparison. **C**, **D.** GSEA plots for the hallmark Inflammatory Response and TNFA Signaling via NFKB, respectively, obtained after analyzing gene expression of PMN-MDSCs and M-MDSCs. * *P* <0.05, ** *P* <0.01, a*** *P* <0.001

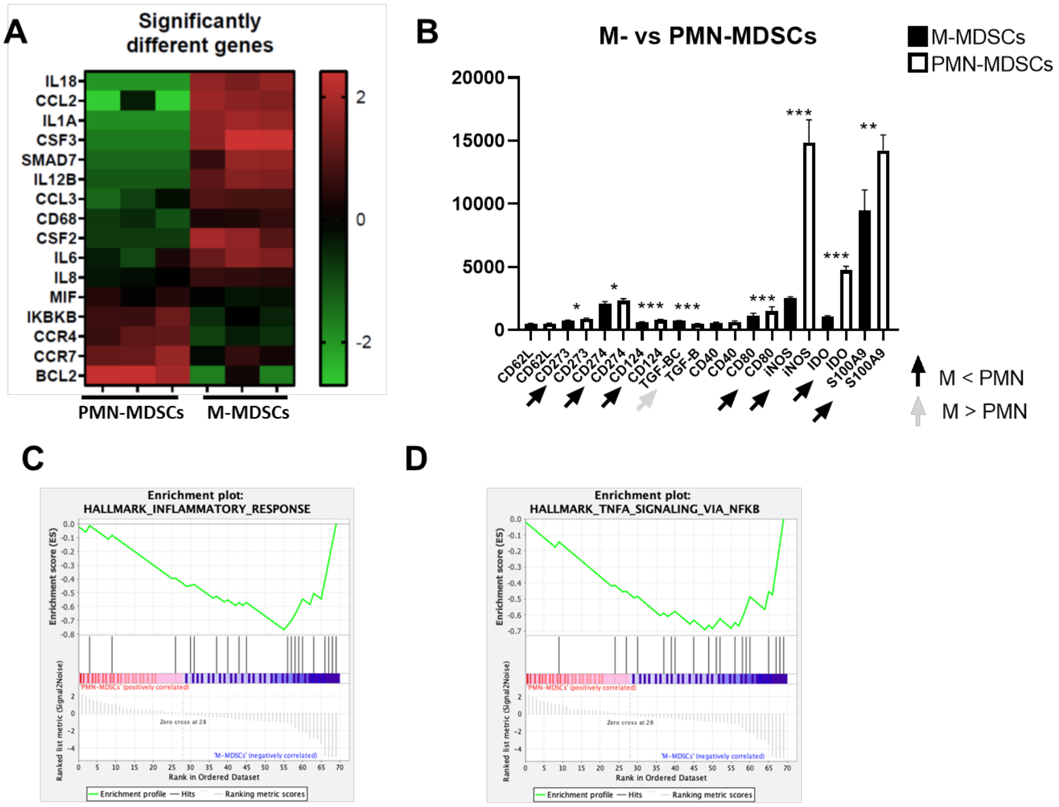

Figure S4: GSEA hallmark TNFA signaling via NFKB gene set

GSEA plot for the hallmark TNFA Signaling via NFKB obtained after analyzing gene expression of mMDSCs and i-mMDSCs from 3 paired samples evaluated by real time PCR in a set of 92 immune-related genes.

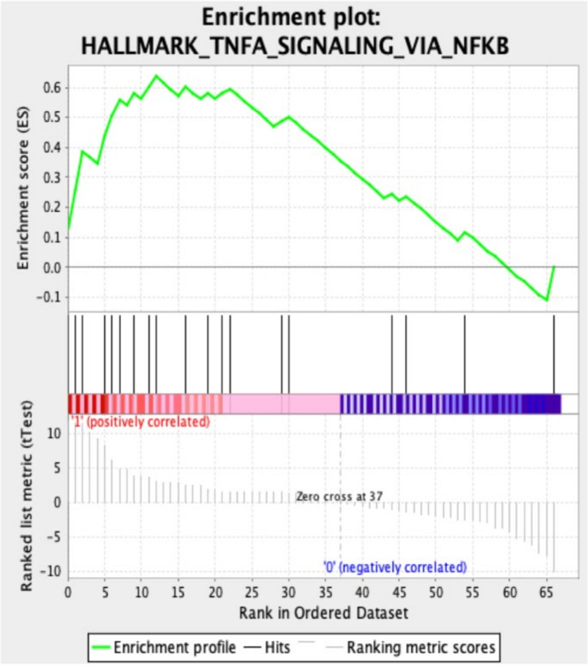

Figure S5: Flow cytometry marker and gating strategies to identify T cells and T-cell subsets.

Flow cytometry strategy for the identification T-cell subsets **A**. Since it is well documented that CD3, CD4, and CD8 levels are reduced on those cells responding to PMA plus ionomycin stimulation[1], we have gated on all T cells (**Figure S3A**, far left) and then on all CD4⁺ T cells (**Figure S3A**, second from left) to adjust for this phenomenon and to best evaluate our findings. **B**. FACS sorting of naïve CD4⁺ T cells.

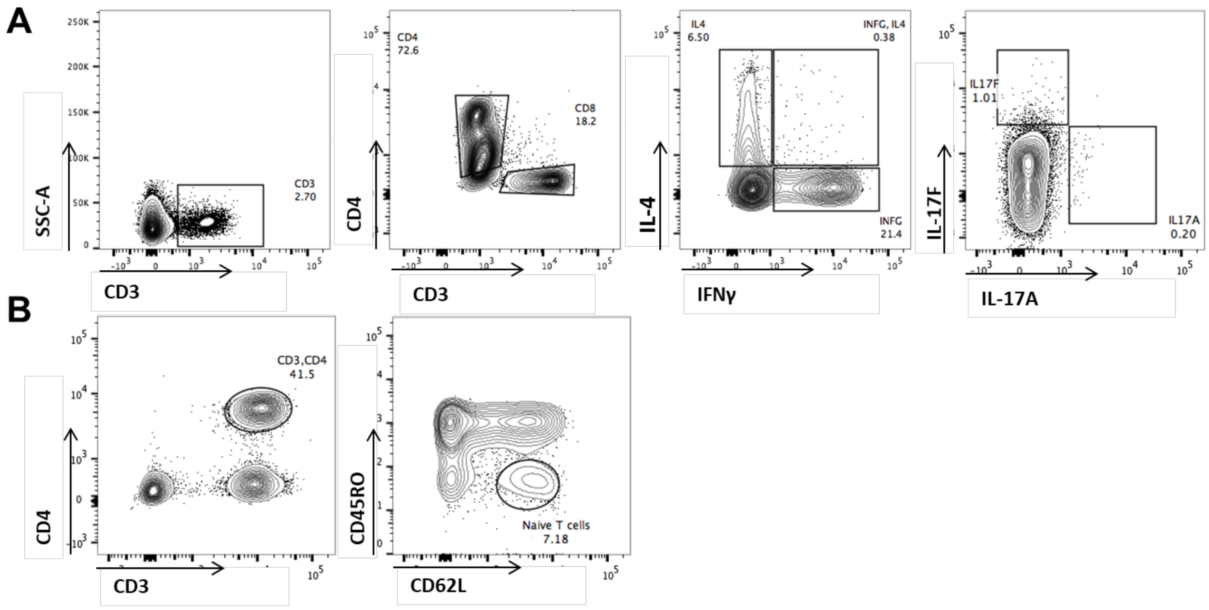

Figure S6: T-cell and T-cell subset numbers in the blood CLL patients and HCs.

A. Absolute numbers of peripheral blood $CD3^+$, $CD4^+$, and $CD8^+$ T cells in 55 CLL patients and 12 HCs. **B**. Absolute peripheral blood counts of cytokine-producing $CD4^+$ T cells in 22 CLL and 7 HCs. Bars represent the median. *P* values: * <0.05, ** <0.01, and ***<0.001.

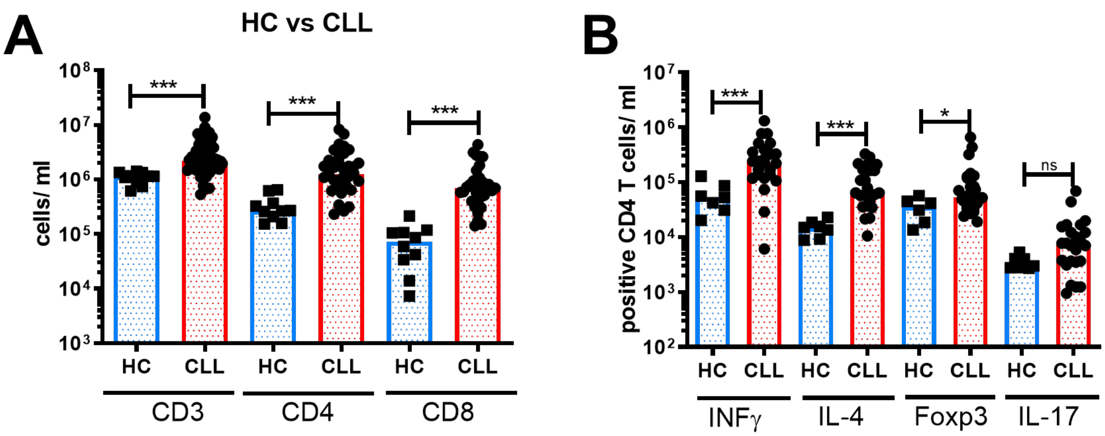

Figure S7: Correlation of MDSC- and T-cell numbers.

Significant correlations exist when comparing T cell (**I**) and major T-cell subset ($CD4^{+}$, $CD8^{+}$) numbers with MDSCs (**A-C**) and MDSC subsets (**D-F**) (n=55) and with $CD4^{+}$ subsets (**G and H**) (n=18).

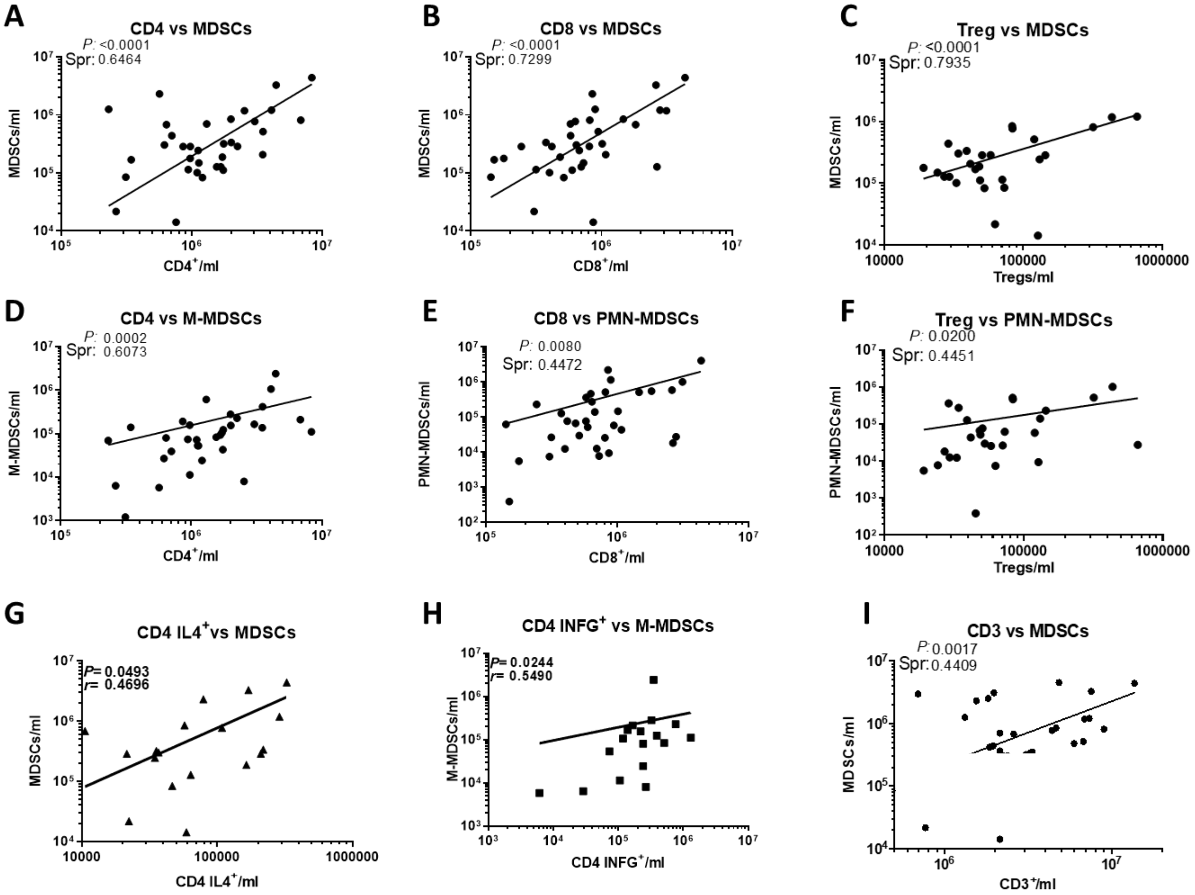

Figure S8: Correlation of MDSC- and T-cell numbers after 3 months of ibrutinib therapy

Significant correlations were found when comparing T-cell and major T-cell subset (CD4^{+}, CD8^{+}) numbers with mMDSC subsets (**A-E**) (n=16) and gMDSCs (**F-I**) (n=16).

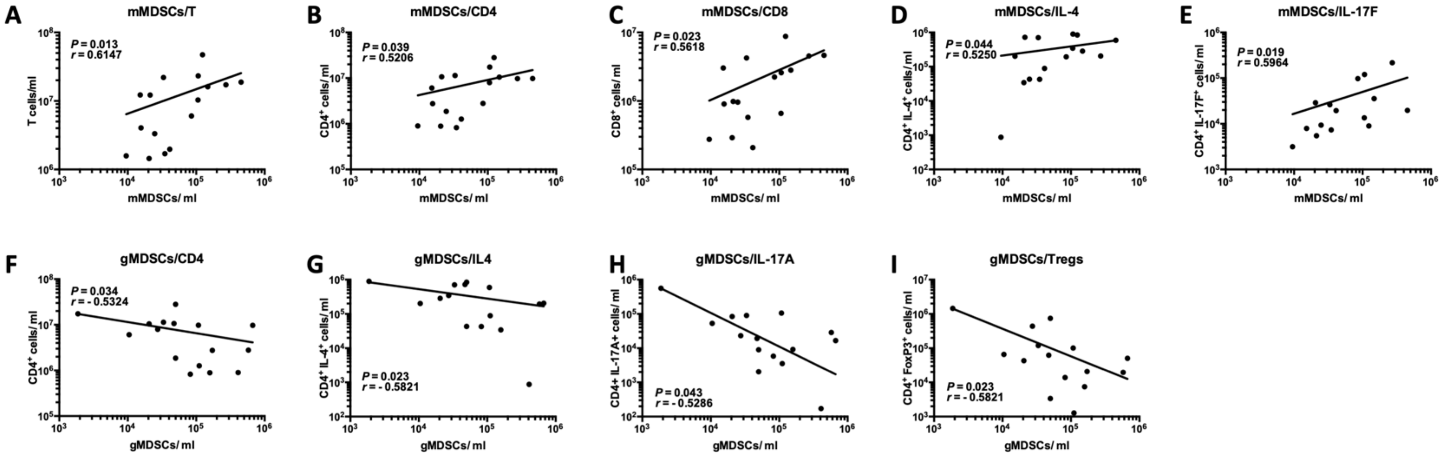

Figure S9: Evolution of Th-cell counts during initial Ibrutinib therapy

Relative CD4⁺ T-cell subset numbers from 16 patients prior to initiating (Pre) and after 1, 2 and 3 months on ibrutinib treatment. Statistical analyses reflect comparisons between pre-treatment and the different time points for a specific population. Dots correspond to median and error lines to interquartile range and continued lines to the connection of the median values. *P* values: * <0.05.

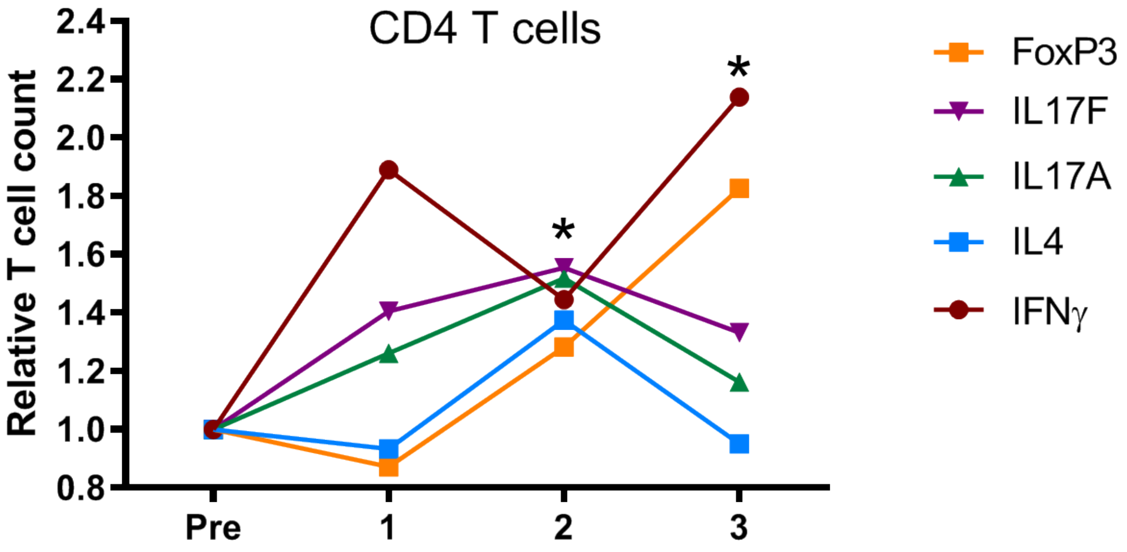

Figure S10: Effect of cryopreservation on MDSC subtype-induced T-cell suppression.

PBMCs were collected from the same patients at two time points (Day 0 and Day 7) and processed the same way each time. The Day 0 sample was cryopreserved in liquid nitrogen until Day 7. At the time of the acquisition of the second sample (Day 7), the first set was thawed and then both FACS-sorted to obtain MDSCs of the two types and $CD4^+$ T. The T-cell suppression assay was performed as described in Material and Methods.

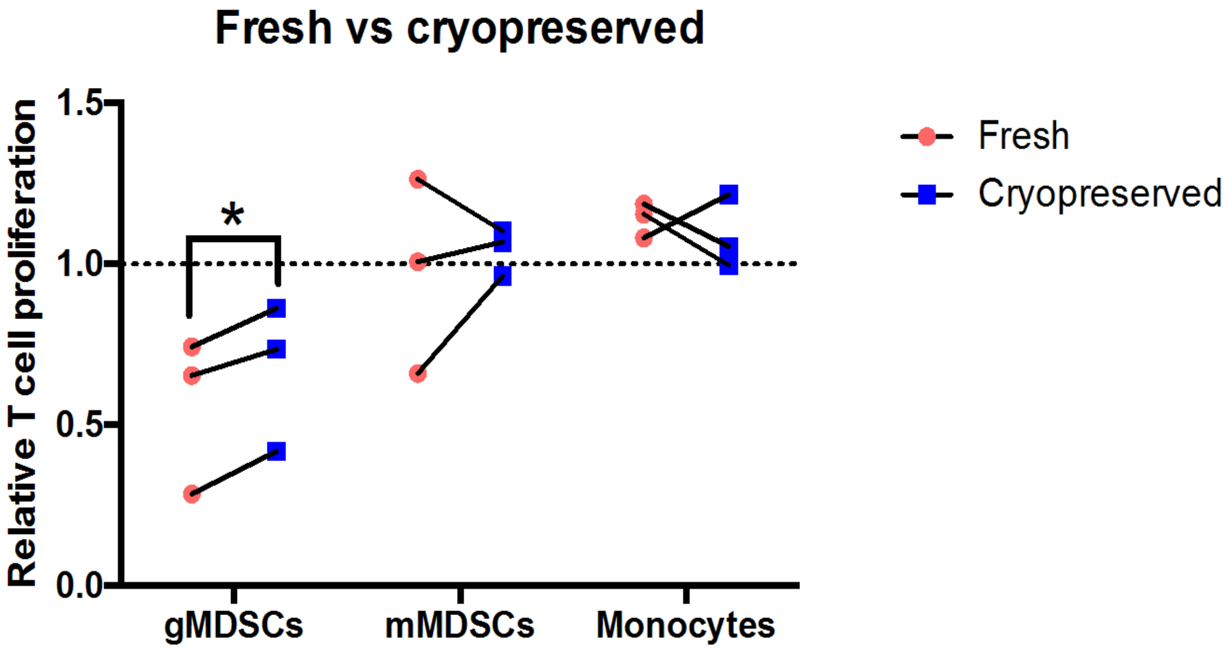

References

1. Kelly K, Shortman K, Scollay R. The surface phenotype of activated T lymphocytes. *Immunol Cell Biol* 1988 Aug; **66 (Pt 4):** 297-306.
